# Pattern of Antibiotic Dispensing at Pharmacies According to the WHO Access, Watch, Reserve (AWaRe) Classification in Bangladesh

**DOI:** 10.3390/antibiotics11020247

**Published:** 2022-02-14

**Authors:** Md. Ariful Islam, Zubair Akhtar, Md. Zakiul Hassan, Sukanta Chowdhury, Md. Mahbubur Rashid, Mohammad Abdul Aleem, Probir Kumar Ghosh, Syeda Mah-E-Muneer, Shahana Parveen, Md. Kaousar Ahmmed, Md. Shakil Ahmed, Ahamed Khairul Basher, Anik Palit, Md Abdullah Al Jubayer Biswas, Zobaid Khan, Khaleda Islam, Nitish Debnath, Mahmudur Rahman, Fahmida Chowdhury

**Affiliations:** 1 Infectious Diseases Division, International Centre for Diarrhoeal Disease Research, Bangladesh (icddr,b), Dhaka 1212, Bangladesh; zakhtar@icddrb.org (Z.A.); zhassan@icddrb.org (M.Z.H.); sukanta@icddrb.org (S.C.); mahbubur.rashid@icddrb.org (M.M.R.); drmdaleem@icddrb.org (M.A.A.); probir@icddrb.org (P.K.G.); mahe@icddrb.org (S.M.-E.-M.); shahana@icddrb.org (S.P.); kaousar@icddrb.org (M.K.A.); shakil.statru@gmail.com (M.S.A.); Khairul.Basher@icddrb.org (A.K.B.); anik.palit@icddrb.org (A.P.); jubayer.biswas@icddrb.org (M.A.A.J.B.); mrahman@globalhealthdev.org (M.R.); fahmida_chow@icddrb.org (F.C.); 2 Nuffield Department of Medicine, University of Oxford, Oxford OX1 2JD, UK; 3 School of Population Health, University of New South Wales (UNSW), Sydney, NSW 1466, Australia; 4 Fleming Fund Country Grant to Bangladesh, DAI Global, LLC, House 3, Road 23B, Gulshan 1, Dhaka 1212, Bangladesh; Zobaid_Khan@dai.com (Z.K.); Khaleda_Islam@dai.com (K.I.); nitish_debnath@dai.com (N.D.); 5 Global Health Development, EMPHNET, 69 Mohakhali, Dhaka 1212, Bangladesh

**Keywords:** antibiotics, dispensing pattern, pharmacy, AWaRe classification, Bangladesh

## Abstract

The WHO Essential Medicines List Access, Watch, and Reserve (AWaRe) classification could facilitate antibiotic stewardship and optimal use. In Bangladesh, data on antibiotic dispensing in pharmacies according to the AWaRe classification are scarce. We aimed to explore antibiotic dispensing pattern in pharmacies according to the WHO AWaRe classification to aid pharmacy-targeted national antibiotic stewardship program (ASP). From January to July 2021, we interviewed drug-sellers from randomly selected pharmacies and randomly selected customers attending the pharmacies. We collected data on demographics and medicines purchased. We classified the purchased antibiotics into the Access, Watch, and Reserve groups among 128 pharmacies surveyed, 98 (76.6%) were licensed; 61 (47.7%) drug-sellers had pharmacy training. Of 2686 customers interviewed; 580 (21.6%) purchased antibiotics. Among the 580 customers, 523 purchased one, 52 purchased two, and 5 purchased three courses of antibiotics (total 642 courses). Of the antibiotic courses, the Watch group accounted for the majority (344, 53.6%), followed by the Access (234, 36.4%) and Reserve (64, 10.0%) groups. Approximately half of the antibiotics (327/642, 50.9%) were purchased without a registered physician’s prescription. Dispensing of non-prescribed antibiotics was higher in the Access group (139/234, 59.4%), followed by Watch (160/344, 46.5%) and Reserve (28/64, 43.8%) groups. These findings highlight the need to implement strict policies and enforce existing laws, and pharmacy-targeted ASP focusing on proper dispensing practices to mitigate antimicrobial resistance in Bangladesh.

## 1. Introduction

In 2019, the World Health Organization (WHO) listed antimicrobial resistance (AMR) as one of the top ten threats to global health [1]. AMR poses a serious threat to global public health, particularly in low- and middle-income countries (LMICs) [1,2]. Irrational use of antibiotics contributes to the emergence of AMR [1]. A growing number of infections are becoming harder to treat as the bacteria that were supposed to be killed by antibiotics are developing different resistance patterns to the medication [3]. Importantly, resistance to the most commonly available antibiotics has been increasing sharply in recent years as a direct consequence of irrational use of antibiotics [4,5]. An estimated 700,000 people die of AMR each year worldwide [6]. This number is predicted to increase sharply, to as many as ten million deaths annually by 2050 if urgent measures are not taken to support rational use of antibiotics [7]. The appropriate use of antimicrobial agents could potentially reduce the emergence of antibiotic resistance [8]. Without harmonized and urgent action from countries in all income brackets, the world is headed for a post-antibiotic era in which common infections could once again kill [9].

In LMICs, drug sellers at pharmacies play an important role in healthcare services by providing health advice and medications, including antibiotics, for common illnesses [10]. One of the main reasons for AMR in LMICs is over the counter (OTC) sale of antibiotics at pharmacies [11,12,13]. Pharmacy is often the first and only source of healthcare outside home for a majority of population in LMICs [10]. However, most antibiotics in pharmacies are sold without a registered physicians’ prescription, resulting in irrational use and overuse of antibiotics [14,15,16]. According to the National Drug Policy in Bangladesh, there should be a ”Grade C pharmacist” in the pharmacy for dispensing drugs and drug sellers are prohibited from dispensing any antibiotics without a registered physician’s prescription [17]. A study in Bangladesh reported, compared to physicians, drug sellers at pharmacies were twice as likely to provide treatment to the population despite the lack of any formal training in Bangladesh [18]. Despite the alarming increase in AMR, practice of irrational drug prescription and OTC sale of antibiotics across different regions is continuously increasing [19]. Antibiotic stewardship programs (ASPs) should be implemented to improve antibiotic dispensing practices at pharmacies. 

Improving the use of antibiotics through ASP is one of the key interventions essential to limit further emergence and spread of antibiotic-resistant microorganisms [20]. In 2017, WHO introduced the Access, Watch, Reserve (AWaRe) classification to assist the development of ASPs at local, national, and global levels and to reduce AMR. To emphasize the importance of rational use of antibiotics, the WHO classified antibiotics into three groups: the Access group of antibiotics, which have low resistance potentials and are used for first-line or second-line therapies; the Watch group of antibiotics used only with specific indications because of higher resistance potentials; and the Reserve group of antibiotics, which should only be used as a last resort when all other antibiotics have failed. In October 2019, the AWaRe classification was updated and reformed as a classification database [20]. In September 2021, the AWaRe classification was further updated to include additional antibiotics that were not previously classified [21]. The WHO’s 13th General Program of Work (GPW) specifies the country-level target of at least 60% of total consumed antibiotics in the Access group by 2023. This indicator was included to monitor the access to essential medicines and progress toward universal health coverage [20]. 

In alignment with the WHO’s Global Action Plan guidelines, Bangladesh prepared a National Action Plan (NAP) in 2017 for containing AMR [22]. However, implementation of the NAP and enforcement of existing government directions to restrict antibiotic use remains insufficient [23,24,25]. It is estimated that Bangladesh has 118,901 retail pharmacies, of which almost 50% of these are unlicensed [26]. Several studies reported the situation has deteriorated in terms of both the rational use and dispensing of antibiotics without a physician’s prescription in Bangladesh and in many parts of the world [27,28,29]. A recent study jointly conducted by the Directorate General of Drug Administration (DGDA), Bangladesh, and the WHO reported in 2021 that the sale of antibiotics increased by 31%, and most antibiotics were sold without a registered physician’s prescription [30]. A report on mapping the antimicrobial supply chain in Bangladesh showed that in terms of sales, most (54%) of the top ten antibiotics belonged to the Watch and 39% in the Access groups [31]. A recent study in Bangladesh on suspected cases of COVID-19 with severe acute respiratory infections (SARI) reported multiple antibiotics were prescribed to a number of individuals with a higher proportion of Watch group (86%), followed by the Access and Reserve groups [32]. However, there is scarcity of data on antibiotic dispensing from pharmacies according to the WHO AWaRe classification in Bangladesh; and such baseline data at pharmacy level would allow policymakers to develop ASP for drug sellers and more effectively monitor antibiotic consumption. Baseline country-level data are important regarding the implementation of ASPs to control 60% consumption of Access group antibiotics as specified by GPW. This study was conducted to fill these knowledge gaps by collecting relevant data on antibiotic dispensing by drug sellers at pharmacies in Bangladesh. We aimed to estimate the proportion and pattern of antibiotic dispensing at pharmacies according to the AWaRe classification. These findings would aid policymakers in revising effective policies, guidelines, and designing interventions to reduce inappropriate dispensing of antibiotics at pharmacies and thus lowering the AMR occurrence.

## 2. Results

### 2.1. Characteristics of Drug Sellers and Their Perspectives on Antibiotics Sales

Among the 128 participating pharmacies, 98 (76.6%) were licensed. All the drug sellers (N = 128) interviewed were males and had an average age of 37 (±10) years [±standard deviation (±SD)]. The median years of working experience of the drug sellers was 10 years [interquartile range (IQR: 6–20 years)]. In total, 61 (47.7%) drug sellers had reportedly completed an accredited professional healthcare-related certification course (Pharmacy Certificate Registration Course), which is a requirement of the Government of Bangladesh (GoB) for operating a pharmacy. In total, 116 (90.6%) drug sellers were aware of government rules on antibiotic sale and, 97 (75.8%) were aware that antibiotics cannot be sold without a prescription of a registered physician (Table 1).

### 2.2. Characteristics of the Customers Attending the Participating Pharmacies

We interviewed 2686 customers attending the 128 pharmacies. The mean number of customers interviewed at each pharmacy was 21 (range: 13–30). The median age of the customers was 38 years (IQR: 29–50 years), and 78.6% were males. The median years of schooling of the customers was 9 years. A total of 883 (32.9%) customers purchased medicine with a physician’s prescription; 633 (24.7%) on recommendation by the drug seller, 718 (26.7%) on recommendation by family members, friends, and traditional healers, and 422 (15.7%) by self-prescription (themselves), respectively (Table 1).

### 2.3. Purchase of Antimicrobials including Antibiotics 

Among the 2686 customers, 679 (25.3%) purchased antimicrobials. Among them, 580 (21.6%), 91 (3.4%), 7 (0.3%), and 1 (0.04%) customers purchased antibiotic, antiparasitic, antifungal, and antiviral drugs, respectively. Among those who purchased antibiotics, 304 (52.4%) purchased antibiotics without a physician’s prescription (Table 2). Of the 580 customers who purchased antibiotics, 523 (90.2%) purchased one, 52 (9.0%) purchased two, and 5 (0.9%) purchased three courses of antibiotics, respectively (642 courses in total).

### 2.4. Most Frequently Purchased Antibiotics

Of the 642 courses of antibiotics purchased, the most frequently purchased antibiotics were cefixime (109, 18.8%), azithromycin (97, 16.7%), metronidazole (82, 14.1%), ciprofloxacin (55, 9.5%), cefuroxime (48, 8.3%), flucloxacillin (42, 7.2%), and amoxicillin (35, 5.5%). The number of drugs accounting for 90% of drug use (DU90%) was 15 (Table 3).

### 2.5. Antibiotics Purchased According to the WHO AWaRe Classification

**Figure 1:**
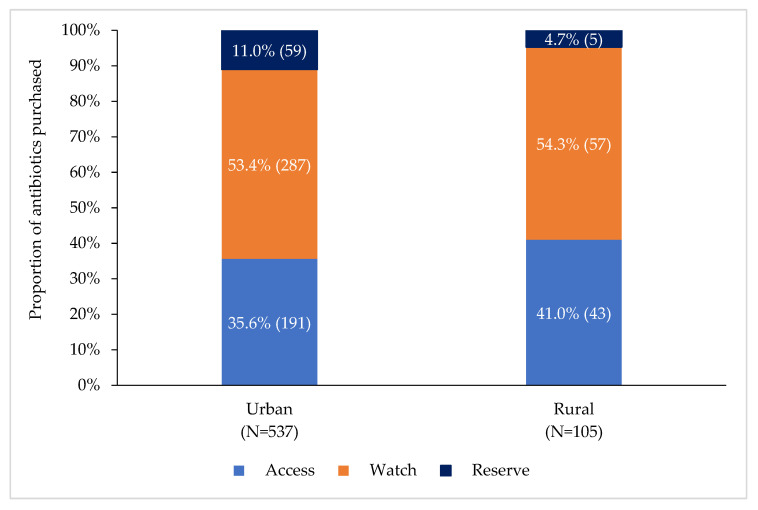
Proportion of antibiotics purchased at rural and urban pharmacies according to WHO AWaRe antibiotic classification, Bangladesh, January–July 2021.

Of the 642 courses of antibiotics purchased, 234 (36.4%), 344 (53.6%), and 64 (10.0%) were from the Access, Watch, and Reserve groups, respectively. Seven antibiotics accounted for 80.1% of total dispensing. These included three antibiotics from the Access group (metronidazole [14.1%], flucloxacillin [7.2%], and amoxicillin [5.5%]) and four from the watch group (cefixime [18.8%], azithromycin [16.7%], ciprofloxacin [9.5%], and cefuroxime [8.3%]) (Table 3). Dispensing of non-prescribed antibiotics was higher in the Access group (139/234, 59.4%), followed by that in the Watch (160/344, 46.5%) and Reserve (28/64, 43.8%) groups (Table 3). Dispensing of antibiotics from the Access, Watch, and Reserve groups accounted for 191 (35.6%), 287 (53.4%), and 59 (11.0%), respectively, in urban pharmacies and for 43 (41.0%), 57 (54.3%), and 5 (4.7%), respectively, in rural pharmacies (Figure 1).

## 3. Discussion

Our study found that the majority of dispensed antibiotics were from the Watch group, and one in ten of the dispensed antibiotics were from the Reserve group, which is quite alarming. There is a scarcity of data on the pattern of antibiotic dispensing in pharmacies in Bangladesh according to the WHO AWaRe classification; however, a recent report showed that a high proportion (86%) of Watch group antibiotics were used for the suspected cases of COVID–19 with SARI [33]. A recently published article on mapping the antimicrobial supply chain in Bangladesh showed that most (54%) of the top ten antibiotics in terms of sales belonged to the Watch group, followed by 39% in the Access group, which is comparable to our study findings [31]. However, the WHO recommends that Access group should comprise at least 60% of the national antibiotic consumption to support AMR mitigation [20].

We observed dispensing of a higher proportion of Watch group antibiotics, which is consistent with the observations in most LMICs including Pakistan, according to an analysis of pharmaceutical sales data from 76 countries [33]. Our study findings showed that approximately 36% of the dispensed antibiotics belonged to Access group, which is similar to the results in the multicounty pharmaceutical sales report [33]. The report demonstrated that Bangladesh had the third-lowest consumption of Access group antibiotic after Japan and India, which is quite lower than the WHO recommendations [33]. Recent studies from LMICs also showed that a smaller share of antibiotics consumption belongs to the Access group (32.5% in Syria and 40.2% in India) [34,35]. However, both the pharmacy sale report and the study in Syria calculated antibiotic consumption using the defined daily doses (DDD) method, which is different from our calculation, and hence, the frequency level comparison might not be appropriate. Even though, the proportion of antibiotic dispensing in our study is not directly comparable with the proportion reported in previous studies, we believe the overall findings of higher use of the Watch group antibiotics and lower use of the Access group antibiotics in these studies are similar to our findings, and may represent a valid comparison. 

The analysis of the pharmaceutical sales report also showed <1% consumption of the Reserve group antibiotics in the majority of countries, whereas in our study in Bangladesh, approximately 10% of the dispensed antibiotics from a pharmacy were from the Reserve group, which is alarming and needs to be considered at the policy level regarding implementation of ASP [33]. This considerable use of the Reserve group antibiotics in pharmacies could be due to the fact that Reserve group of antibiotics are easily available in pharmacies for OTC sales. In our study, approximately half of the Reserve group of antibiotics were dispensed irrationally by the drug sellers without a registered physician prescription. This also highlights a lack of strict monitoring mechanism. We observed that cefixime (a third-generation cephalosporins), azithromycin (macrolides), and metronidazole (imidazoles) were the frequently dispensed antibiotics at pharmacies in Bangladesh, comprising almost 45% of total antibiotics dispensed. The use of cefixime and azithromycin was similar to the results of a recent mobile survey conducted across Bangladesh during the COVID-19 pandemic. In that survey, azithromycin was found to be the most frequently used antibiotic, followed by cefixime, among those who could recall the names of prescribed antibiotics [36]. The use of cefixime and azithromycin was comparable with survey findings of the pattern of antibiotic dispensing in private pharmacies in Nepal, in which cefixime was found to be the most frequently used antibiotic and azithromycin was ranked sixth in terms of sale [37]. Cefixime and azithromycin belong to the Watch group of antibiotics, which are recommended to be used only in case of a limited group of well-defined syndromes to limit AMR [38].

We noted that approximately half the antibiotics were dispensed without a registered physician’s prescription. This result is consistent with published literature in Bangladesh, which show that a significant proportion of antibiotics were sold without a registered physician’s prescription [27,39,40,41,42]. Our findings are comparable with an estimation of the WHO, which showed that >50% of the antibiotics worldwide were sold without a physician’s prescription [43]. Studies from LMICs also showed that a significant proportion of non-prescribed antibiotics were dispensed [44,45,46,47]. A recent report from six LMICs showed that self-medication with antibiotics was found to be widespread in Vietnam, followed by that in Bangladesh and Ghana, but was less common in Mozambique, South Africa, and Thailand [48]. A systematic review on antibiotic dispensing showed that antibiotics were frequently dispensed without a registered physician’s prescription in many countries; which markedly affects the emergence of AMR [49]. We also found that, without a registered physician’s prescription approximately 59% of the dispensed antibiotics belonged to the Access group, nearly half to the Watch and Reserve groups; this raises a flag to immediately adopt and strengthen a “prescription-only from formal providers” policy for antibiotic purchase [42] Our findings emphasize the urgent need for enforcement of regulations for prescription-only antibiotics, implementation of a pharmacy-based antimicrobial monitoring program, and training programs for drug sellers on proper antibiotic dispensing practices. Though our study did not explore the underlying factors for non-prescribed sales of antibiotics, studies have documented poor access to healthcare facilities, financial benefits to both customers and drug sellers, dependency on pharmaceutical companies, limited knowledge of drug sellers, and lack of enforcement policies as the major contributing factors that need to be addressed at a policy level [50,51].

Our study demonstrated that 77% of the participating pharmacies were operating with a license issued by the GoB. This percentage was considerably higher than that reported in a 2015 baseline pharmacy study, in which almost 50% of pharmacies were unlicensed, indicating a positive change with respect to licensed pharmacies over the year [26]. Our survey observed that only 50% of the drug sellers had completed pharmacy training (Pharmacy Certificate Registration Course), which is a prerequisite to operate a pharmacy. The percentage of drug sellers with pharmacy training found in this study was considerably higher than that reported in a previous study performed in Dhaka city, which showed that only 11% drug sellers had pharmacy training [52]. These findings indicate that over the years, a higher proportion of drug sellers with completed pharmacy training are operating pharmacies and thus adhering to the national pharmacy licensing policy. However, to comply with Bangladesh’s 2005 National Drug Policy, enforcement of laws and regulations for the pharmacy licensing policy to operate a pharmacy should be prioritized and appropriate ASPs should be implemented for drug sellers to mitigate AMR.

The current study has produced several important results; however, it has some limitations, which should be considered while interpreting our study findings. Our survey was conducted in one district of each of the eight divisions and five out of nine divisional city corporations in Bangladesh, and only a small number of pharmacies were surveyed because of resource constraints. Additionally, our survey was limited to pharmacies located >100 m away from of any medical college or hospital to identify the antibiotic dispensing practices followed by typical drug sellers. Moreover, the study was conducted during the COVID-19 pandemic; thus, drug dispensing patterns may have been different from the pre-pandemic period.

## 4. Materials and Methods

### 4.1. Study Sites and Study Population

**Figure 2:**
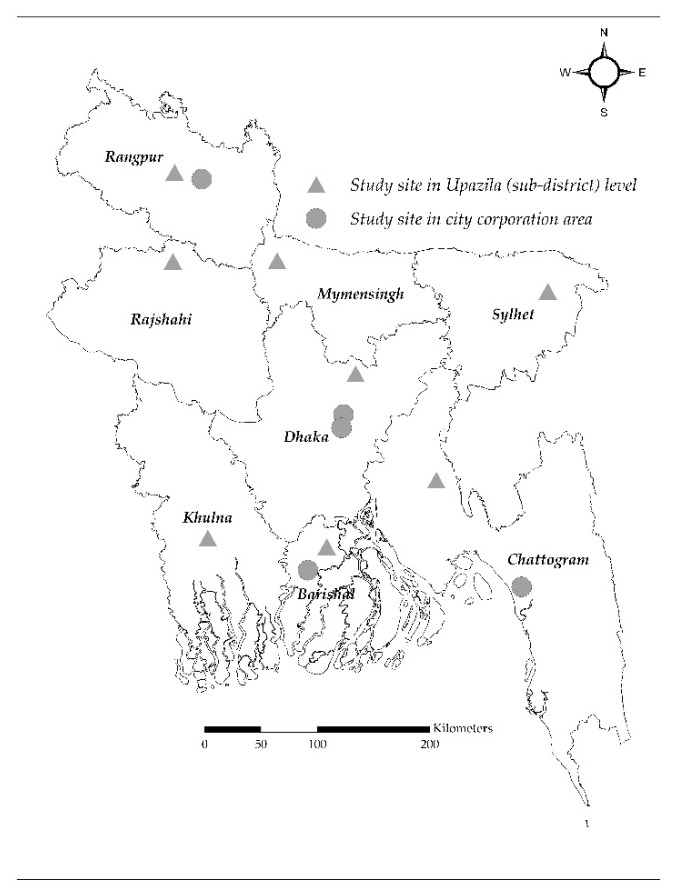
Map of Bangladesh showing the study sites.

This study was conducted as a component of a National Point Prevalence survey (PPS) on antimicrobial use in humans and animals in Bangladesh from January to July 2021. The broader objective of the PPS was to estimate the proportion of antimicrobial use among patients seeking healthcare at hospitals, customers at pharmacies, for commercial chicken, and aquaculture in Bangladesh. The survey of this component was conducted at randomly selected 112 urban and 16 rural pharmacies located in eight divisions of the country. Considering resource constraints, these pharmacies were randomly selected from eight districts (one district from each of the eight divisions) and five city corporations (selected from the nine divisional city corporations). At a district level, first, we randomly selected eight Upazilas (sub-districts), one from each of the eight districts. Then, we selected 96 pharmacies, 12 from each of the Upazilas from the listed pharmacies in the respective study areas using simple random sampling. We selected ten pharmacies from the headquarter areas of each Upazila and one pharmacy from each of the randomly selected two Unions (the smallest rural administrative and local government entity) under each Upazila (Figure 2, Figure S1). In city corporation areas, we randomly selected 32 pharmacies from five divisional city corporations. First, we grouped the nine divisional city corporation areas into two categories: large city corporation areas (Dhaka North, Dhaka South, Chattogram, Khulna and Rajshahi) and small city corporation areas (Barishal, Ranjpur, Sylhet and Mymensingh). For the large city corporation category, Dhaka North and Dhaka South city corporations were purposely selected to include pharmacies from the capital city of Bangladesh, Dhaka. For this category, Chattogram city corporation was randomly selected. For the small city corporation category, Barishal city corporation and Ranjpur city corporation were randomly selected. After selecting 5 city corporations, we randomly selected eight wards from each of the selected city corporations, except Dhaka North and Dhaka South city corporations. For Dhaka North and Dhaka South city corporation, 4 wards were randomly selected from each of the city corporations. Finally, 32 pharmacies were selected from the listed pharmacies in the respective study areas using simple random sampling; eight pharmacies were selected from Dhaka North and Dhaka South city corporations (4 from each of the city corporations) and 24 pharmacies were selected from Chattogram city corporation, Ranjpur city corporation, and Barishal city corporation (8 from each of the city corporations). Pharmacies located at the Union level were defined as rural pharmacies and those located at Upazila (sub-district) headquarter and city corporation area were defined as urban pharmacies (Figure 2, Figure S1). The sites in the district level covering Upazila headquarters and Unions represents both rural and urban areas; however, sites in city corporation area covered urban area only, representing large cities. The pharmacies were also categorized as licensed (government approved) or unlicensed (not government-approved) pharmacies. Pharmacy licenses are provided to drug sellers by DGDA, Government of Bangladesh, when they have completed at least a ”Grade C pharmacist” certificate degree (i.e., three-month course), which grants them permission to legally operate a pharmacy and dispense drugs. Pharmacies that dispensed medication were included, and those located within a 100 m radius of government and private medical colleges and hospitals were excluded to identify the antibiotic dispensing practices by typical drug sellers. Field staff, comprised of four Field Research Assistants and a Senior Field Research Officer, enrolled the pharmacies for the study, obtaining written informed consent of the drug seller. If a selected pharmacy refused to participate, then the field staff included the next closest pharmacy in accordance with the eligibility criteria.

### 4.2. Selection of Participants

#### 4.2.1. Drug Seller

In our study, drug seller was defined as a person working at a pharmacy who recommends and sells drugs, and may or may not have any formal training in pharmacy practice [53]. From the randomly selected pharmacies, the field staff identified drug sellers (one from each of the pharmacies) who spent the most time attending customers each day for conducting a face-to-face interview (Figure S1).

#### 4.2.2. Customers

The field staff explained to the drug sellers that they would wait in the pharmacy from 10:00 am to 10:00 pm on the day of the survey to identify adult customers who purchased medicines for themselves or for a sick relative with or without a physician’s prescription. Accordingly, we enrolled one customer attending each of the pharmacies every 20 min using systematic random sampling. The field staff selected one customer in each of 20-min time slots for conducting a face-to-face exit interview. If there were no customers in the specific 20-min time slots, none were enrolled from the following time slots to supplement the enrollment. Customers aged >18 years were enrolled, considering adult customers could provide reliable survey information.

### 4.3. Data Collection

Separate structured questionnaires were used to collect data from drug sellers and customers after obtaining written informed consent. Both questionnaires included information on the demographic characteristics of drug sellers and customers. Additionally, the questionnaire for drug sellers included relevant information regarding antibiotic dispensing and customers’ questionnaire included the name of the purchased medicine and medicine purchasing practices.

### 4.4. Statistical Analysis

The data were illustrated using frequency and percentage for all the categorical variables related to the drug sellers and customers attending the pharmacies. Continuous variables were summarized using mean, standard deviation (SD), median, and interquartile range (IQR) based on the distribution of the variables. We used Chi-square test, Fisher’s exact test and Z-score test to compare the differences in antibiotics dispensed with and without registered physicians’ prescriptions where the *p*-value < 0.05 was considered statistically significant. The primary outcome of interest was purchased antibiotics, which was determined based on the drugs purchased by the customers. Antimicrobial drugs including antibiotic, antiviral, antiparasitic, and antifungal drugs were identified from the purchased drugs. Antibiotics were further classified into Access, Watch, and Reserve groups according to the 2021 WHO AWaRe classification [21]. The proportion of antibiotic dispensing was measured among participants who were interviewed. Among those who purchased antibiotics, the frequencies of course of the antibiotics were listed. A course of antibiotics was defined as a specific type of antibiotic received by the customer during a pharmacy visit. If a customer received two different types of antibiotics in the same visits, we counted this as two courses of antibiotics. This total course of antibiotics was used as the denominator for calculating the proportion of antibiotics purchased for each AWaRe category. We also calculated the number of drugs accounting for 90% of drug use, using drug utilization 90% (DU90%) index [54]. Statistical analyses were performed using the Stata 13 software (StataCorp. 2013. Stata Statistical Software: Release 13. College Station, TX: StataCorp LP.).

## 5. Conclusions

We found that Watch group of antibiotics accounted for the majority of the dispensed antibiotics, and approximately half of the antibiotics were dispensed without a registered physician’s prescription. Our study findings highlight the need for the implementation of a strict policy and enforcement of existing law to restrict non-prescription sale of antibiotics and pharmacy-targeted antibiotic stewardship interventions in Bangladesh. Integration of the WHO AWaRe classification into the NAP on AMR in Bangladesh, implementing pharmacy-targeted interventions, and emphasizing mass awareness among the general population, including drug sellers and customers that might contribute to mitigating AMR in the country.

## Figures and Tables

**Table 1:** Characteristics of drug sellers and customers attending pharmacies in Bangladesh during January–July, 2021.

Drug Sellers Characteristics	*n* = 128
	***n*** **(%)**
Age in years (mean ± SD *)	36.8 ± 10
Male	128 (100.0)
**Education level (years of schooling)**	
6–10	22 (17.2)
11–12	45 (35.2)
13–16	51 (39.8)
16+	10 (7.8)
Pharmacy certificate registration course completed	61 (47.7)
Working experience at pharmacy in years, median, (IQR **)	10 (6-20)
Awareness of government policy on antibiotics sale	116 (90.6)
Could correctly state government directives on antibiotics sale	97 (75.8)
Pharmacy license shown by drug seller	98 (76.6)
**Customers’ Characteristics**	***n* = 2686** ***n* (%)**
Median age in years, (IQR **)	38 (29–50)
Male	2118(78.9)
Female	568 (21.1)
**Education level (years of schooling)**	
No formal education	351 (13.1)
1–5	540 (20.1)
6–10	929 (34.9)
11–12	386 (14.4)
13–16	365 (13.6)
16+	115 (4.3)
**Medicine purchased for**	
Customer himself/ herself	1391 (51.8)
Family member	1260 (46.9)
Others	35 (1.3)
**Medicine purchased with the advice from**	
Physician	883 (32.9)
Drug seller	663 (24.7)
Own-decision/self-medication	422 (15.7)
Others (family members, friends and traditional healers)	718 (26.7)
**Antibiotics purchased with the advice from (*n* = 580)**	
Physician	276 (47.6)
Drug seller	172 (29.7)
Own-decision/self-medication	22 (3.8)
Others (family members, friends and traditional healers)	110 (19.0)

* SD: Standard Deviation; ** IQR: Interquartile Range.

**Table 2:** Antimicrobials purchased by customers from pharmacies in Bangladesh, January–July, 2021.

Antimicrobial Purchased	Customers	Purchased with Prescription	Purchased without Prescription	*p*-Value
	*n* = 2686	*n* = 883	*n* = 1803	
	*n* (%)	*n* (%)	*n* (%)	
**Antimicrobial**	679 (25.3)	314 (35.6)	365 (20.2)	<0.001
**Antibiotics** *	580 (21.6)	276 (31.3)	304 (16.9)	<0.001
**Number of antibiotics bought**				
One antibiotic	523 (90.2)	241 (87.3)	282 (92.8)	0.066
Two antibiotics	52 (9.0)	31 (11.2)	21 (6.9)	
Three antibiotics	5 (0.9)	4 (1.4)	1 (0.3)	
**Antiparasitic**	91 (3.4)	36 (4.1)	55 (3.1)	0.167
**Antifungal**	7 (0.3)	2 (0.2)	5 (0.3)	
**Antiviral**	1 (0.04)	0 (0)	1 (0.1)	
**Other than antimicrobial drugs bought**	2007 (74.7)	569 (63.8)	1,438 (79.8)	<0.001

* A total of 580 customers purchased 642 courses of antibiotics.

**Table 3:** Antibiotics purchased by customers attending pharmacies according to the WHO Access, Watch, Reserve (AWaRe) classification, Bangladesh, January–July, 2021.

Number	Generic Name of Antibiotics	WHO AWaRE Classification	Antibiotics *	With Prescription	Without Prescription	*p*-Value
			*n* = 642	*n* = 315	*n* = 327	
			*n* (%)	*n* (%)	*n* (%)	
**Drug Utilization 90% (DU90%) 1–15**			**588 (91.6%)**	283 (89.8%)	305 (93.3%)	
1	Cefixime	Watch	109 (18.8)	61 (22.1)	48 (15.8)	0.052
2	Azithromycin	Watch	97 (16.7)	40 (14.5)	57 (18.8)	0.17
3	Metronidazole	Access	82 (14.1)	34 (12.3)	48 (15.8)	0.231
4	Ciprofloxacin	Watch	55 (9.5)	27 (9.8)	28 (9.2)	0.814
5	Cefuroxime	Watch	48 (8.3)	34 (12.3)	14 (4.6)	0.001
6	Flucloxacillin	Access	42 (7.2)	19 (6.9)	23 (7.6)	0.752
7	Amoxicillin	Access	35 (5.5)	6 (2.2)	29 (9.5)	<0.001
8	Linezolid	Reserve	19 (3.3)	6 (2.2)	13 (4.3)	0.155
9	Polymyxin B	Reserve	18 (3.1)	12 (4.4)	6 (1.8)	0.1
10	Cefradine	Access	18 (3.1)	6 (2.2)	12 (3.7)	0.219
11	Levofloxacin	Watch	15 (2.6)	8 (2.9)	7 (2.3)	0.652
12	Faropenem	Reserve	15 (2.6)	9 (3.3)	6 (1.8)	0.329
13	Doxycycline	Access	13 (2.2)	5 (1.8)	8 (2.6)	0.505
14	Amoxycillin + Clavulanic acid	Access	12 (2.1)	10 (3.6)	2 (0.7)	0.012
15	Phenoxymethylpenicillin	Access	10 (1.7)	6 (2.2)	4 (1.3)	0.428
Others 16–35			54 (8.40%)	32 (10.20%)	22 (6.70%)	
16	Chloramphenicol	Access	8 (1.4)	3 (1.1)	5 (1.6)	0.565
17	Colistin	Reserve	8 (1.4)	5 (1.8)	3 (0.9)	0.395
18	Gentamicin	Access	6 (0.9)	3 (1.1)	3 (0.9)	0.905
19	Ceftriaxone	Watch	4 (0.7)	3 (1.1)	1 (0.3)	0.271
20	Tedizolid	Reserve	4 (0.7)	4 (1.5)	0 (0)	0.035
21	Sulfamethoxazole + Trimethoprim	Access	3 (0.5)	0 (0)	3 (0.9)	0.098
22	Cefaclor	Watch	3 (0.5)	2 (0.7)	1 (0.3)	0.507
23	Clarithromycin	Watch	3 (0.5)	2 (0.7)	1 (0.3)	0.507
24	Tetracycline	Access	2 (0.3)	0 (0)	2 (0.7)	0.177
25	Erythromycin	Watch	2 (0.3)	1 (0.4)	1 (0.3)	0.945
26	Meropenem	Watch	2 (0.3)	0 (0)	2 (0.7)	0.177
27	Cefalexin	Access	1 (0.2)	1 (0.4)	0 (0)	0.294
28	Chloramphenicol + Dexamethasone phosphate	Access	1 (0.2)	1 (0.4)	0 (0)	0.294
29	Clindamycin	Access	1 (0.2)	1 (0.4)	0 (0)	0.294
30	Cefotaxime	Watch	1 (0.2)	1 (0.4)	0 (0)	0.294
31	Cefpodoxime	Watch	1 (0.2)	1 (0.4)	0 (0)	0.294
32	Cefpodoxime Proxetil	Watch	1 (0.2)	1 (0.4)	0 (0)	0.294
33	Ceftazidime	Watch	1 (0.2)	1 (0.4)	0 (0)	0.294
34	Neomycin sulfate	Watch	1 (0.2)	1 (0.4)	0 (0)	0.294
35	Streptomycin	Watch	1 (0.2)	1 (0.4)	0 (0)	0.294
Total			642 (100%)	315 (100%)	327 (100%)	

* A total of 580 customers purchased 642 courses of antibiotics. Note: Total number of different types of antibiotics in Access, Watch, and Reserve groups were 234 (prescribed vs. non-prescribed: 95 vs. 139; *p*-value = 0.001) 344 (prescribed vs. non-prescribed: 184 vs. 160; *p*-value = 0.016), and 64 (prescribed vs. non-prescribed: 36 vs. 28; *p*-value = 0.237), respectively.

## Data Availability

The data presented in this survey are available on reasonable request from icddr,b’s research administration through the corresponding author. The data are not publicly available due to privacy restrictions and icddr,b policy.

## Supplementary Materials

The following are available online at https://www.mdpi.com/article/10.3390/antibiotics11020247/s1, Figure S1: Study sites and study population selection flowchart
