# Supplementary figures and images for: Pattern of Antibiotic Dispensing at Pharmacies According to the WHO Access, Watch, Reserve (AWaRe) Classification in Bangladesh
Source: Antibiotics (Basel). 2022 Feb 14;11(2):247. doi: 10.3390/antibiotics11020247 (PMC8868217; doi:10.3390/antibiotics11020247)

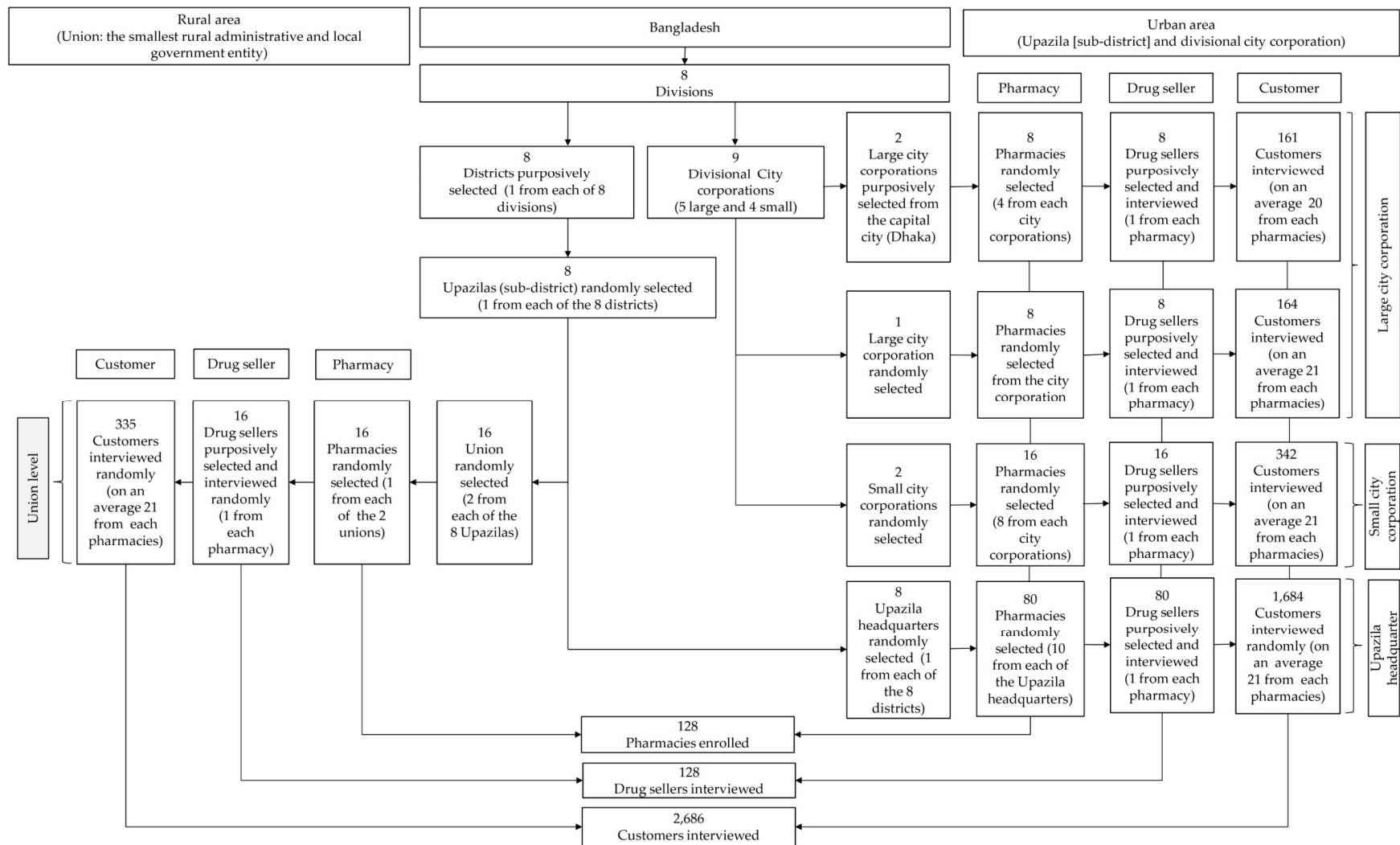

**Figure S1.** Study sites and study population selection flowchart

Supplement: Supplementary file 1 [file antibiotics-11-00247-s001.zip › antibiotics-1563293-supplementary.pdf]
